# A fearful scourge to the penguin colonies: Southern giant petrel (*Macronectes giganteus*) predation on living Magellanic penguins (*Spheniscus magellanicus*) may be more common than assumed

**DOI:** 10.1002/ece3.11258

**Published:** 2024-04-24

**Authors:** Eric L. Wagner, Ginger A. Rebstock, P. Dee Boersma

**Affiliations:** ^1^ Center for Ecosystem Sentinels, Department of Biology University of Washington Seattle Washington USA; ^2^ Wildlife Conservation Society Bronx New York USA

**Keywords:** *Macronectes giganteus*, Magellanic penguin, natural history, predation, southern giant petrel, *Spheniscus magellanicus*

## Abstract

Southern giant petrels (*Macronectes giganteus*) are important consumers that range across the oceans throughout the southern hemisphere. In Argentina, previous studies have shown they eat primarily pinnipeds and penguins, which they are assumed to scavenge, although there are occasional anecdotes of them attacking living penguins. Here we describe a predation attempt by a trio of southern giant petrels on a molting adult Magellanic penguin (*Spheniscus magellanicus*) at the large colony at Punta Tombo, Argentina. We relate giant petrel attendance patterns at the colony to the penguins' phenology, showing how giant petrel numbers rise with the increasing prevalence of vulnerable penguins. We suggest that living penguins—both fledglings and adults—may constitute a more seasonally significant proportion of the giant petrel diet than previously assumed, and their capture may represent a specialized predation technique.

## Graphical abstract

Southern giant petrels are thought to be primarily scavengers in Argentina. We report a predation attempt by several giant petrels on an adult Magellanic penguin. By situating it within giant petrel attendance patterns at the colony, we suggest predation of living penguins might not be as rare as presumed.

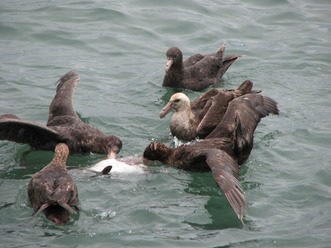

## 1 INTRODUCTION

**FIGURE 1:**
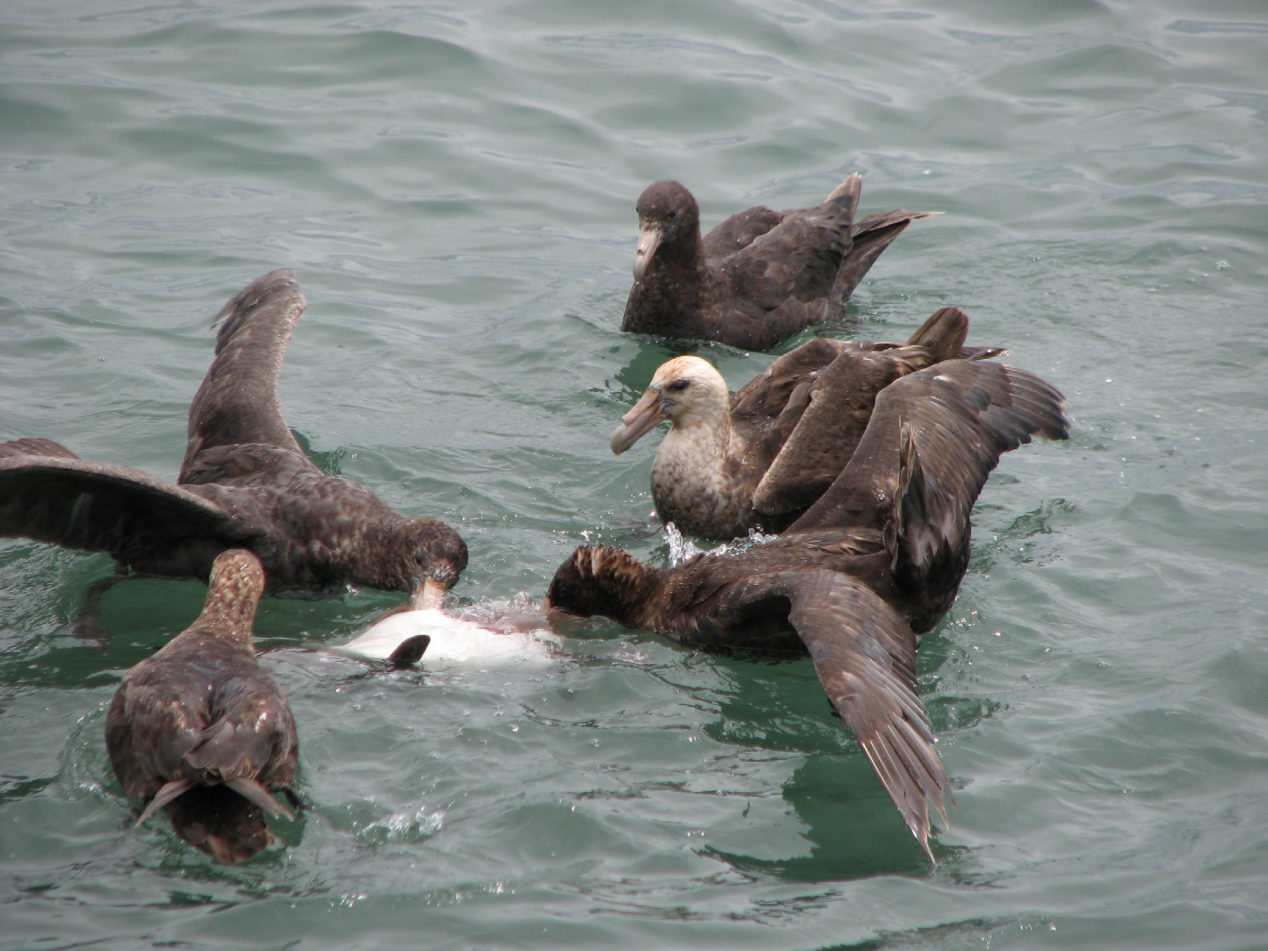
Five southern giant petrels (*Macronectes giganteus*) feed on a dead juvenile Magellanic penguin (*Spheniscus magellanicus*) in the waters off of Punta Tombo, Argentina, site of a large penguin colony. Note how the two birds actively eating the penguin have their wings outstretched, and the bird on the right further has raised and fanned its tail. Note also the plumage variations among the birds, from wholly brown (likely juvenile or immature) to one with a whitish head (an adult). Photograph by Dee Boersma.

The southern giant petrel (*Macronectes giganteus*) is a generalist predator and scavenger that ranges widely in the southern hemisphere (Hunter, 1983; Murphy, 1936). In Argentina, giant petrels are thought to feed primarily on cephalopods, although they also eat pinniped and penguin carrion (Copello et al., 2008; Forero et al., 2004, 2005). In the centrally‐located Province of Chubut, where giant petrels have two major breeding colonies, the penguin carrion on which they subsist comes almost exclusively from Magellanic penguins (*Spheniscus magellanicus*) (Figure 1), which also have several major colonies on the coastal mainland and some offshore islands (García‐Borboroglu et al., 2022).

Living penguins are assumed to make up only a small part of the giant petrel diet, and then perhaps only for some individual giant petrels, i.e., as a specialist technique (Copello et al., 2008). Even when giant petrels do predate living penguins, they are assumed not to target adults consistently (Boersma et al., 2013; Copello et al., 2008). These assumptions are based primarily on stable isotope studies (e.g., Copello et al., 2008), however, which cannot show whether a food source was living or dead.

Although there are anecdotal reports of giant petrels predating adult Magellanic penguins (e.g., Conway, 1971; Harris, 1998; Wagner, 2018), and we have witnessed such events over the years, the act is generally assumed to be so rare as to be aberrant, and no formal descriptions exist (but see, e.g., Punta & Herrera, 1995 and Descamps et al., 2005 for accounts of giant petrels preying on blue‐eyed cormorants (*Phalacrocorax atriceps*) and king penguins (*Aptenodytes patagonicus*), respectively).

Here, we report an observation of three southern giant petrels trying to prey on a molting adult Magellanic penguin at the penguin breeding colony at Punta Tombo, Argentina. While their attempt was unsuccessful, it demonstrates the technique giant petrels use to try to kill adult Magellanic penguins on the water. It also illustrates the conflicts that can occur when an individual who may specialize in a predation technique is interfered with by a conspecific that either does not employ the technique or may be less practiced at it (e.g., Ceia & Ramos, 2015). Finally, we situate the giant petrels' attempted predation within the species' seasonal attendance patterns and penguin phenology at Punta Tombo. Given that we have seen giant petrels herding penguins on the beach during the molt on several occasions, and dead adults on the beach that appeared killed at sea, we argue that living penguins may be an underappreciated prey source for southern giant petrels in Argentina and elsewhere.

## 2 METHODS

### 2.1 Study site and species' phenological patterns

We have followed a marked population of Magellanic penguins at their colony at Punta Tombo, Argentina (44°03′ S, 65°13′ W) since 1982 (Boersma et al., 1990). The colony is large but declining, with approximately 120,000 breeding pairs (Rebstock et al., 2016). The penguins' breeding season begins in early September when males arrive to claim and defend nests after overwintering at sea (Boersma et al., 1990). Females arrive a couple of weeks later and lay a clutch of two eggs. Chicks begin to hatch in late November. Both parents then feed their chicks, and those that survive start to fledge in late January (Wagner & Boersma, 2019). Parents will feed their chicks through February, but then typically stop to prepare for their molt (Boersma et al., 2013).

One‐year‐old juveniles start to arrive at the colony in January to molt into adult plumage. The adult molt cycle begins in March with a foraging trip that might last up to 3 weeks (Boersma et al., 2013). When the adults return, they are landbound for an additional ~19 days while they molt all feathers simultaneously (Boersma et al., 2013). During this time, individuals frequently go to the beaches to preen, drink, bathe, or socialize (Boersma et al., 2013).

Southern giant petrels are present during the penguins' breeding season at Punta Tombo through April, when adult penguins molt (Boersma et al., 2013). During the molt, petrels often patrol the beach and surf zone on the wing, swim in the water close to shore, or rest on the beach. On the beach, they sometimes herd groups of penguins while looking for individuals in poor condition. They also frequently scavenge penguins that starved to death. When several giant petrels are present, they compete for a carcass by mantling over it or charging to displace those that are actively consuming it (e.g., Bretagnolle, 1988).

Southern giant petrels do not solely scavenge at Punta Tombo, however. When chicks fledge, petrels will often sit on the water within 100 m of the beach, presumably to capture fledglings as they leave the colony. We have observed petrels attacking and sometimes killing fledglings many times over the years, both on the beach and in the water. On the beach, giant petrels target weak fledglings, biting them on the neck and sometimes starting to tear their cloacas open even though the fledglings are still alive.

### 2.2 Attempted predation on molting adult Magellanic penguin

On 19 April 2023, E. Wagner and G. Rebstock observed a predation attempt by a giant petrel on a molting adult Magellanic penguin. Below we describe the behaviors of the giant petrel that initiated the attack, and those of two giant petrels that later arrived and interfered with the first giant petrel. We also describe the behavior of the penguin that was attacked. This adds to several giant petrel attacks we have either witnessed or seen evidence of throughout the project.

### 2.3 Magellanic penguin peak fledging date and weight

We determined the peak penguin fledging date at Punta Tombo to compare with southern giant petrel attendance patterns using a series of morning counts made from an elevated boardwalk, where an observer counted fledglings as they left the colony to go to sea. We also counted the number of southern giant petrels in the vicinity, whether on land or water, or flying. We began these counts in mid‐January, before chicks started to fledge, and counted every other day until almost all the fledglings had left the colony (i.e., the morning counts yielded zero fledglings, although a few chicks may still have been present here and there). The date when the observer counts the greatest number of fledglings per hour is considered the peak fledging date. To find the peak fledging date for all years, we converted the calendar date to Julian Day and took the average ± the standard deviation.

We also looked at the weights of fledging chicks. Every year since 2007 (excluding 2011 and 2020), we weighed fledglings as they left the colony to start their northward migration. Fledglings were caught in the mornings from late January through late February, usually between 0700 and 0900 Argentina time (ART). We captured and weighed 71.3 ± 30.5 fledglings per year (range, 20–137). To see if there was a within‐season temporal trend in fledgling weight, we used a linear mixed model (LMM) in a Bayesian framework, with fledging weight as the response variable and days since September 1 as a fixed effect. The year was a random effect. The full model for observation *i* in year *j*[*i*] was therefore

$$\;y_{i}\sim N\left(\mu_{i},\sigma \right)$$

$$\mu_{i}=\alpha +a_{j\left[i\right]}+\beta_{1}D_{i}$$

$$a_{j}\sim N\left(0,\sigma_{\text{Year}}\right)$$

where α is the intercept, $a_{j\left[i\right]}$ is the random effect of year, and $D_{i}$ is number of days since 1 September.

### 2.4 Beach counts for southern giant petrels to determine colony attendance patterns

Since 1986, we have done a regular beach count from the same elevated boardwalk to determine penguin beach‐attendance patterns throughout the season. These counts took place in the early evening, and were done every 5 or 6 days until the end of the field season. In addition to all the penguins within roughly 100 m of the observation point, we counted southern giant petrels whether they were on land or water, or flying, as well as other potential predators, such as South American sea lions (*Otaria flavescens*).

To see how southern giant petrel counts changed from September through April, and whether there were peaks of abundance rather than a simple linear trend, we used a Poisson generalized additive mixed model (GAMM) in a Bayesian framework. The response variable was the count of southern giant petrels from either the morning or evening counts, with two main effects: days since 1 September (i.e., the start of the field season) with a cubic regression spline, and count time of day (morning or evening). The year was a random effect. The full model for observation *i* in year *j*[*i*] was therefore

$$y_{i}\sim \text{Pois}\left(e^{\pi_{i}}\right)$$

$$\log\left(\pi_{i}\right)=\alpha +a_{j\left[i\right]}+f_{1}D_{i}+\beta_{1}T_{i}$$

$$a_{j}\sim N\left(0,\sigma_{\text{Year}}\right)$$

where α is the intercept, $a_{j\left[i\right]}$ is the random effect of year, is days since 1 September with a cubic regression spline smooth function ($f_{1}$), and $T_{i}$ is the count time of day.

All Bayesian models were created in the Stan computational framework in R, using the *rstanarm* package for the LMM and the *brms* package for the GAMM (Bürkner, 2017). The *rstanarm* package allows fitting many of the most commonly applied regression models using Markov Chain Monte Carlo (MCMC)—in this case Hamiltonian Monte Carlo (Monnahan et al., 2017). The prior distribution for the first model was selected to be weakly informative, with N(0, 5) priors on the intercept and regression coefficients. To improve convergence and guard against overfitting in the second model, we likewise specified weakly informative conservative priors, with N(0, 3) priors on the intercept and regression coefficients. We ran four independent chains for 5000 iterations after a warmup of 1000 iterations for both models. MCMC convergence was assessed using the potential scale reduction factor diagnostic ($\hat{R}$ ≤ 1.05; Gelman et al., 2013) and visual inspection of chains.

## 3 RESULTS

### 3.1 Observation of predation attempt on molting adult Magellanic penguin

During a morning beach count at Punta Tombo on 19 April 2023, at 0920 ART, E. Wagner and G. Rebstock observed a predation attempt from the elevated observation point. Two groups of penguins had gathered on the beach about 150 m from each other. The group that was closest to the observers had more than 100 penguins; the one farther away had ~30 penguins. All were adults, and most had either finished or nearly finished molting, although a few still had substantial remnants of old plumage. Some of the penguins in this last group were in poor condition, with prominent keels and an overall emaciated appearance.

**FIGURE 2:**
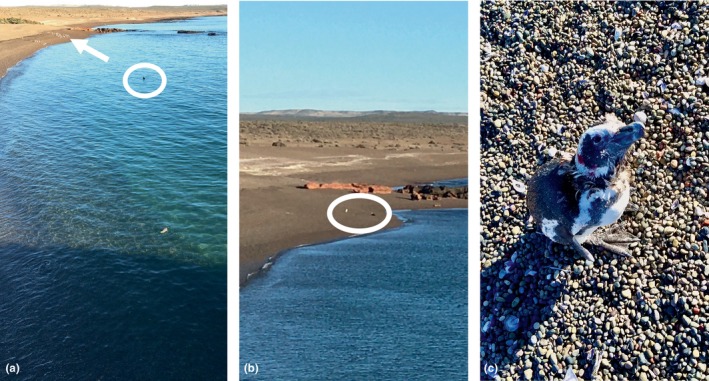
(a) The first southern giant petrel (GP1, circled) approaches a more distant group of penguins (white arrow). (b) The second southern giant petrel (GP2, right‐most dot in the white circle) sits near the injured penguin (left‐most dot) on the beach during the predation attempt, preventing it from entering the water. Shortly after this picture was taken, the penguin advanced on the petrel in a whirling fashion and drove it away. (c) The penguin following the described predation attempt. Note that it is emaciated and the side of its face is bloody. Photographs A and B by Eric Wagner; photograph C by Ginger Rebstock.

A dark southern giant petrel flew in and landed on the water ~15 m from the shore. Based on its plumage it was likely an immature (Carlos & Voisin, 2008, but note that in the *soladeri* subspecies, which breeds on the Falkland Islands / Islas Malvinas and Argentine coasts, a few breeders remain brown until their plumage lightens after a few years.) The penguins became alert but did not leave the beach. The giant petrel (GP1) paddled slowly between the two groups of penguins, causing individuals either to cluster more tightly on the beach or run into the water and swim a short distance from shore, presumably for safety (Figure 2a).

After approaching each penguin group two or three times, the giant petrel paddled toward the smaller, more distant group, then left the water and walked up the beach with its wings outstretched. In response, about half the penguins raced farther up the beach toward a ridge that separates the beach from the colony proper, while the other half ran into the sea. The group in the sea then split further, with 10 birds swimming farther offshore toward open water, and five swimming laterally along the shore over an outcropping of rock that was mostly submerged due to the tidal height. The giant petrel focused on this smaller group, swimming in broad circles and seeming to contain the penguins in the area over the submerged rocks. Finally, the petrel zeroed in on one penguin that was visibly weaker than the others and began to pursue it in earnest.

The giant petrel tried to grab the penguin with its bill. Perhaps due to the topography of the submerged rocks, or perhaps because the penguin had not finished molting, it did not swim directly away from the giant petrel to open water but instead circled it, coming up for quick, porpoising breaths every few seconds. The giant petrel snapped at the penguin several times when it surfaced but missed. Finally, after about a minute, the giant petrel succeeded in catching and holding the penguin by the neck/face region.

The giant petrel then attempted to drown the penguin, spreading its wings and plunging the penguin's head underwater. It also maneuvered its body so it could stand atop the penguin. The penguin visibly struggled throughout—sometimes a flipper flailed from the water—and every so often succeeded in raising its head before the giant petrel thrust it underwater again. The giant petrel was able to hold the penguin's head underwater for up to ~20 seconds at a time, but never longer. This lasted for nearly 5 min. Finally, the penguin's struggles seemed to become less vigorous, and the giant petrel relaxed its posture. It appeared that the penguin's strength was waning.

At this time, a second giant petrel (GP2) flew in and landed near GP1. It swam near GP1 and the penguin with its wings outstretched and its tail raised, an agonistic posture (Bretagnolle, 1988). Within a minute, a third giant petrel (GP3) landed, too, swimming a short distance from the other two without posturing. The three petrels were easily distinguishable by their plumage: GP1 was entirely dark, GP2 had a whitish head more typical of older birds (Carlos & Voisin, 2008), and GP3 was intermediate.

Although neither of the two newly arrived giant petrels directly interfered with GP1's attempts to drown the penguin, less than a minute after their arrival, the penguin broke free of GP1's grip. GP1 attempted to grab it, but the penguin snapped at GP1 and drove it back. The penguin did not attempt to swim away from the three giant petrels, however. It appeared exhausted, floating on the water and panting heavily, even though the giant petrels were only a few meters from it.

GP1 moved away from the penguin, yielding to the more socially dominant GP2; GP3 hung back but did not leave right away. GP2 advanced on the penguin, but its approach was less assertive than GP1's had been, and the penguin fended the giant petrel off with lunges and bill snaps. After about 2 min, the penguin had the strength to swim back to the beach and try to walk. It was unsteady, though, stumbling and falling to its belly a couple of times before simply standing in place and swaying from side to side.

GP2 waited a few minutes before following the penguin to the beach. (During this time, GP1 and GP3 drifted farther and farther away from the beach before they both eventually flew away.) GP2 left the water and walked toward the penguin. The penguin lunged and snapped at GP2, who backed away. GP2 approached again, the penguin again lunged and snapped, and GP2 once more retreated and sat down on the beach (Figure 2b). The penguin huffed and did head turns—a general antagonistic display (Boersma et al., 2013). The standoff lasted for about 2 min before the penguin began to spin in circles and obliquely advance on the giant petrel, snapping its bill. At this, the giant petrel flew away. The penguin stood alone and then walked up the beach perhaps 20 m to the ridge, where it rested.

The entire incident was finished by 0947. A few minutes later, E. Wagner and G. Rebstock left the observation point and approached the penguin to observe it more closely (Figure 2c). The bird had nearly finished molting but was clearly in poor condition. We visually assessed the penguin to be a male based on his bill depth (Boersma et al., 2013), but he was otherwise very thin, with a prominent keel and spine, both of which are signs of progressive emaciation. Gashes on the right side of his head were bleeding, if no longer so profusely. His flippers were still trembling, but in addition to having enough energy to walk up the beach, he also brayed and lunged at G. Rebstock before continuing over the ridge. Owing to his injuries and obvious distress we did not weigh him. Nor did we follow him, so his final destination—and his ultimate fate—is unknown.

### 3.2 Peak penguin chick fledging, fledging weight, giant petrel colony attendance, and giant petrel attacks

**FIGURE 3:**
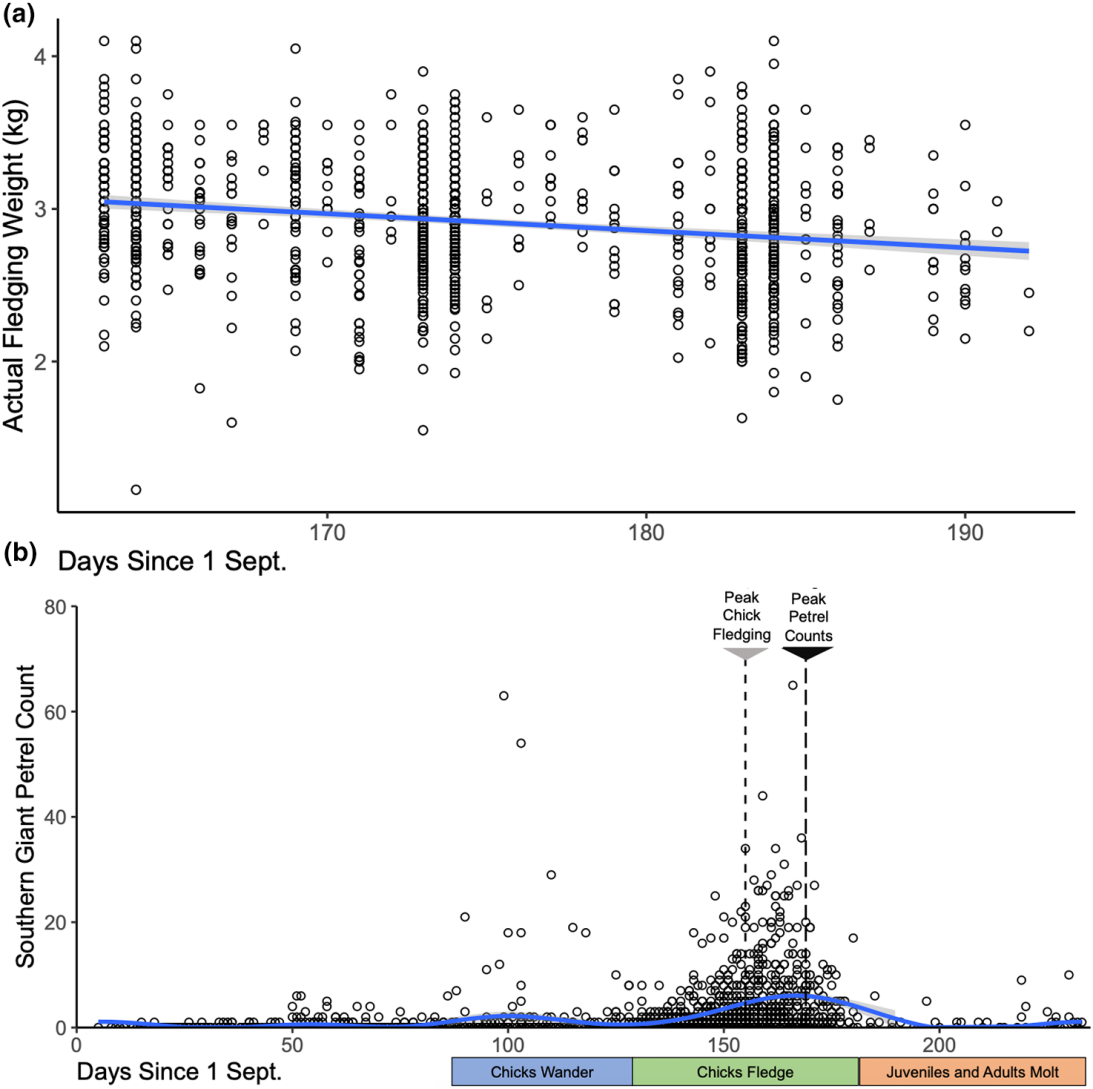
(a) Weights of fledglings captured as they left the colony for the sea. Solid blue line is the linear trend, with shaded gray lines as 95% credible intervals. (b) Counts of southern giant petrels seen during morning and evening observations at the Magellanic penguin colony at Punta Tombo, Argentina. Evening counts were done every 5–6 days from September through April; morning counts were done from mid‐January through mid‐February. Giant petrels were counted if they were on the beach or swimming in the general vicinity of the observation point, or flew past it during the count. The blue line with gray shaded credible intervals shows fit from a Poisson generalized additive model (GAMM). Penguin chick and adult phenologies are shown below the x‐axis, while dashed vertical lines indicate the dates of average peak fledging and giant petrel peak abundance.

The average peak fledging date for Magellanic penguin chicks at Punta Tombo was 3 February ±1 day, with dates ranging from 24 January (in 1992) to 17 February (in 2004). The average peak fledging rate from the morning counts was 38.6 ± 3.01 chicks per hour (range, 8.0–85.4). Fledglings weighed more the earlier they fledged, going from 3.11 ± 0.36 kg in mid‐January to 2.66 ± 0.37 kg by mid‐February (Table 1, Figure 3a).

**TABLE 1:** Average fledging weights as a function of days since September 1 at the Magellanic penguin colony at Punta Tombo, Argentina.

Parameter	Coefficient (95% CI)
Intercept (α)	5.00 (4.45, 5.55)
**Days since 1 September (** $\beta_{1}$ **)**	**−0.012 (−0.008, −0.015)**
$\sigma_{\text{year}}$	0.11 (0.07, 0.18)

*Note*: Coefficient estimates (mean and 95% credible interval [CI] in parentheses) are from a Bayesian linear mixed model. Hierarchical variance component—$\sigma_{\text{year}}$: interannual intercept SD. Main effect in bold is that with a 95% CI that did not cross 0, indicating strong support.

We did 1724 morning‐fledging and evening beach‐count surveys at Punta Tombo from September 1986 through April 2023. Counts of southern giant petrels varied widely, from zero to a high count of 65 individuals seen on 14 February 1987; 63 were also counted on 9 December 2015. Overall, 51 counts had 10 giant petrels or more; of those, 35 counts were in February, nine were in December, five were in January, and one each in November and April. The Poisson GAMM model with petrel count as a function of days since 1 Sept explained 33% of the deviance, with giant petrel attendance having four defined peaks (Table 2, Figure 3b): a first small peak around 22 October (0.4 ± 0.2 petrels per count); a second more substantial peak around 14 December (2.2 ± 0.9 petrels per count); a third—and the largest—peak around 17 February (5.1 ± 1.7 petrels); and finally a fourth, more modest peak around 6 April (1.8 ± 0.9 petrels). Count time of day had a weak effect, with evening counts tending to have fewer petrels than morning counts (Table 2). Note that when morning counts were excluded, the peaks of giant petrel attendance did not change.

**TABLE 2:** Average southern giant petrel counts from morning and evening surveys as a function of days since September 1 and the timing of the survey (morning or evening) at the Magellanic penguin colony at Punta Tombo, Argentina.

Parameter	Coefficient (95% CI)
Intercept (α)	0.18 (−0.10, 0.45)
**Cubic Spline: Days since September 1 (** $f_{1}$ **)**	**1.64 (0.96, 2.86)**
*Time of Survey: PM (* $\beta_{1}$ *)*	*−0*.*07 (−0*.*14*, *0*.*01)*
$\sigma_{\text{year}}$	0.81 (0.62, 1.08)

*Note*: Coefficient estimates (mean and 95% credible interval [CI] in parentheses) are from a Bayesian Poisson generalized additive mixed model. Hierarchical variance component—$\sigma_{\text{year}}$: interannual intercept SD. Main effect in bold is one whose 95% CI did not cross 0, indicating strong support; main effect in italics is one whose 80% CI did not cross 0, indicating weaker support.

During the four decades of our long‐term study, field researchers have observed 16 instances where one or more southern giant petrels either killed or attempted to kill a penguin or multiple penguins (e.g., herding them on the beach). Of those, four attacks were on fledglings, three were on juveniles, six were on adults, and three had no notes on the penguin's age class. Two attacks were seen in December, and seven apiece in January and February. The witnessed attacks included a pair of giant petrels herding penguins on the beach but not necessarily pursuing one in earnest, a giant petrel drowning an adult penguin ~50 m from shore, a giant petrel attacking a juvenile that was sleeping on the beach, and a giant petrel walking up to an adult penguin of unspecific condition and simply pecking it to death with its bill.

Additionally, we have recorded ~80 instances over the years where we suspected a giant petrel had attacked or killed an adult, juvenile, or fledgling, but could not confirm it because we had not witnessed the act. Evidence for such inferences included but was not limited to a penguin being freshly dead on the beach even though it appeared to be in good condition, or penguins with bloody wounds on their heads that were indicative of being attacked while they were alive.

## 4 DISCUSSION

### 4.1 Predation of living Magellanic penguins may not be rare

The southern giant petrels' predation attempt on the molting adult Magellanic penguin was one of several attacks, both successful and unsuccessful, on live penguins that researchers have observed at Punta Tombo, not only in April 2023, but over the course of our long‐term study. In addition, we have noted dozens of instances where giant petrels were suspected to have attacked living penguins, although the act itself was not witnessed. Southern giant petrels are typically described as scavengers of carrion based both on observation and stable isotope analysis (Copello et al., 2008), but the frequency of these attacks on live penguins suggests that, at least at Punta Tombo, they may not be as rare as is widely presumed.

Our regular morning and evening counts over 35 field seasons have shown that giant petrel attendance at Punta Tombo varies over time, starting from effectively zero at the beginning of the breeding season, when only adult penguins in generally robust condition are present, before increasing in mid‐October when male penguins might have been fasting for a few weeks and are preparing to leave (Boersma et al., 2013). The mid‐December peak coincides with the time that chicks start to wander as a prelude to fledging (Boersma et al., 2013). The peak in February follows the peak chick fledging. Late fledglings are lighter than fledglings that leave earlier, making them easier prey, which likely explains why peak giant petrel attendance was ~2 weeks after peak fledging. The smaller April peak aligned with the period when all the chicks are gone, but many adults are still at the colony, either molting or having just completed their molt (Boersma et al., 1990). Some of these adults are about to start their northward migration; others are in such poor condition that they will not survive.

All of this suggests giant petrels are progressively drawn to the colony by the increased presence of vulnerable prey. Penguin chicks that are out of the guard stage and starving will often go down to the beach (Boersma pers obs.). Once they die, they are usually consumed by scavengers, including kelp gulls (*Larus dominicanus*) and brown skuas (*Stercorarius antarcticus*). The giant petrel will generally displace these two species, though, when it arrives at a carcass (Wagner, 2018). Evening counts tended to have fewer petrels than morning counts, which was because evening counts occurred throughout the season, i.e., at the beginning when there were few petrels, but morning counts were at the heart of the fledging period when more petrels were present.

As predators, both northern (*M*. *halli*) and southern giant petrels are capable of capturing and killing a variety of seabirds and marine mammals in different life stages (e.g., Anderson, 2002; Descamps et al., 2005; Murphy, 1936; Punta & Herrera, 1995; Van Buren, 2012). The giant petrel's beak and associated muscles are well adapted to deliver a strong bite, which is advantageous for killing larger prey, such as penguins (Mazzochi & Carlos, 2022). Northern giant petrels will come into king penguin colonies at night to kill chicks, although co‐occurring southern giant petrels were less likely to engage in these behaviors (Le Bohec et al., 2003). Northern giant petrels will also kill northern rockhopper penguins (*Eudyptes mosleyi*) at sea, but, again, southern giant petrels were less likely to engage in the behavior, and when they did were less likely to be successful (Ryan et al., 2008).

When southern giant petrels do attack and kill living penguins—especially adults—those penguins are typically weak or injured (e.g., Descamps et al., 2005). While they molt, one‐year‐old juvenile and adult Magellanic penguins grow weaker by the day, since they are fasting (Boersma et al., 2013). The molt may thus represent the most opportune time for giant petrels to predate adults. In the predation attempt we observed, the penguin was in poor condition, although not so poor that he could not ultimately fight off the three larger birds.

### 4.2 A specialist technique inconsistently adopted and imperfectly applied

During the observed predation attempt, we noted that the first southern giant petrel, likely an immature, appeared to have subdued the penguin and would likely have drowned it soon had the other two giant petrels not arrived. Although we cannot know the thoughts of one bird or another, the presence of two potential competitors—one of whom was likely an adult and thus socially dominant—appeared to distract the first giant petrel such that the penguin was able to escape. Afterwards, even though the penguin was injured and weak, it was able to fight off two giant petrels. (The third never committed to the attack).

Southern giant petrels in Patagonia show dietary segregation based on sex and, potentially, age (Forero et al., 2005), with males more likely to forage coastally (Copello et al., 2011). Giant petrels also show pronounced sexual dimorphism, with males weighing 40% more than females (Copello et al., 2006), and juveniles and immature birds having largely dark plumage, while adults are lighter and their heads almost white (Harris, 1998). We suggest that the principle consumers of living penguins in Argentina are likely to be younger male giant petrels.

Giant petrels are not known to cooperate when they forage; where there is evidence of sociality in foraging seabirds generally, it is typically in the following of conspecifics to prey patches (e.g., Jones et al., 2018) and perhaps attacking prey schools simultaneously (e.g., Harris et al., 2023). Many giant petrels will congregate to consume a single carcass, however. When they do, they have elaborate displays to establish a kind of rotating hierarchy (Bretagnolle, 1988). In one display, a giant petrel flares its tail and spreads its wings over a carcass. GP2 was engaging in such a display—necessarily modified because it was swimming—when the penguin freed itself from GP1's grip.

When giant petrels have been seen preying on live penguins, it is assumed these might be individual specialists (Copello et al., 2008). Individual of several marine bird species, including giant petrels (e.g., Grohmann Finger et al., 2021), are known to have specialist foraging strategies in specific circumstances or locales (Ceia & Ramos, 2015). Kelp gulls around Peninsula Valdés, for example, specialize in feeding on mother‐calf pairs of southern right whales (*Eubalaena australis*), stripping the skin from the whales' backs (Rowntree et al., 1998). This behavior was first documented in 1972 and has spread so widely that the majority of southern right whales using waters around Peninsula Valdés now have lesions from gull attacks (Azizeh et al., 2021; Fazio et al., 2012).

Such specialization might extend not only to method but also intent. In most accounts of giant petrels predating living adult penguins (e.g., Conway, 1971; Wagner, 2018), it is either unknown or unclear whether the petrel purposefully hunted an adult or merely came across it during the course of foraging. What was notable in this incident, then, was that GP1 deliberately sought to pursue a live adult penguin. Rather than come upon a weakened one by happenstance and subsequently kill it, GP1 moved between two groups of penguins with the apparent aim of flushing, isolating, and killing an adult. The stalking took several minutes, and had to all appearances a kind of protocol. All of this suggests that living penguins could be an important component of southern giant petrel diets, with implications for predator–prey dynamics.

## AUTHOR CONTRIBUTIONS

**Eric L. Wagner:** Conceptualization (lead); data curation (lead); formal analysis (lead); investigation (equal); methodology (equal); writing – original draft (lead). **Ginger A. Rebstock:** Conceptualization (supporting); investigation (equal); methodology (supporting); writing – review and editing (equal). **P. Dee Boersma:** Project administration (lead); supervision (lead); writing – review and editing (equal).

## CONFLICT OF INTEREST STATEMENT

The authors declare no competing interests.

## Data Availability

Data are available on Dryad: https://doi.org/10.5061/dryad.zkh1893ht.
